# A Micropatterned Multielectrode Shell for 3D Spatiotemporal Recording from Live Cells

**DOI:** 10.1002/advs.201700731

**Published:** 2018-01-04

**Authors:** Jordi Cools, Qianru Jin, Eugene Yoon, Diego Alba Burbano, Zhenxiang Luo, Dieter Cuypers, Geert Callewaert, Dries Braeken, David H. Gracias

**Affiliations:** ^1^ imec, Department of Life Sciences and Imaging Kapeldreef 75 3001 Leuven Belgium; ^2^ KU Leuven, Department of Cellular and Molecular Medicine 3000 Leuven Belgium; ^3^ Department of Chemical and Biomolecular Engineering Johns Hopkins University Baltimore MD 21218 USA; ^4^ Centre for Microsystems Technology (CMST) Ghent University and imec Technologiepark – Zwijnaarde 15 9052 Gent Belgium; ^5^ Department of Materials Science and Engineering Johns Hopkins University Baltimore MD 21218 USA

**Keywords:** biosensing, cardiomyocytes, electrophysiology, microelectrode arrays, neuroscience

## Abstract

Microelectrode arrays (MEAs) have proved to be useful tools for characterizing electrically active cells such as cardiomyocytes and neurons. While there exist a number of integrated electronic chips for recording from small populations or even single cells, they rely primarily on the interface between the cells and 2D flat electrodes. Here, an approach that utilizes residual stress‐based self‐folding to create individually addressable multielectrode interfaces that wrap around the cell in 3D and function as an electrical shell‐like recording device is described. These devices are optically transparent, allowing for simultaneous fluorescence imaging. Cell viability is maintained during and after electrode wrapping around the cel and chemicals can diffuse into and out of the self‐folding devices. It is further shown that 3D spatiotemporal recordings are possible and that the action potentials recorded from cultured neonatal rat ventricular cardiomyocytes display significantly higher signal‐to‐noise ratios in comparison with signals recorded with planar extracellular electrodes. It is anticipated that this device can provide the foundation for the development of new‐generation MEAs where dynamic electrode–cell interfacing and recording substitutes the traditional method using static electrodes.

## 1 Introduction

Microelectrode arrays (MEAs) are the preferred method for long‐term studies of electrophysiological phenomena, and have been successfully applied in fundamental neuroscience, drug discovery, safety pharmacology, and neuroprosthetics.^1^, ^2^, ^3^, ^4^ In contrast to the well‐established patch‐clamp technique,^5^ MEAs provide high‐throughput by allowing bidirectional and noninvasive interfacing with a large number of cells in parallel. The most common MEAs are the commercially available “passive” systems, where relatively large metal electrodes are connected to external recording and stimulation units. State‐of‐the‐art MEAs that utilize on‐chip multiplexing architectures provided by integrated circuit (IC) or complementary metal‐oxide‐semiconductor (CMOS) technology can comprise up to 60 000 electrodes enabling highly parallel electrophysiological recordings at subcellular resolution.^4^, ^6^, ^7^, ^8^, ^9^ Regardless of the technology, a common goal is to maximize cell–electrode adhesion and electrical coupling in order to achieve high signal quality. Strategies include selective coating of the electrodes using adhesive biomolecules,^10^ conductive polymers,^11^, ^12^ or carbon nanomaterials.^13^, ^14^, ^15^, ^16^, ^17^

In addition to coatings, the geometry of the electrodes can be engineered to enhance recording performance. While the standard design of MEAs feature flat or pillar‐shaped electrodes, Spira and co‐workers developed gold spine electrodes in which cells engulf mushroom‐shaped 3D protrusions of 1–2 µm diameter.^18^, ^19^ They claim that this shape and engulfment increases seal resistance and localizes ionic channels at the membrane–microelectrode interface. Elsewhere, 3D nanoFET‐based probes coupled into kinked nanowires have been reported.^20^ Using premodified phospholipids, these nanowires were shown to gently penetrate the cell membrane and allowed intracellular recordings of the five characteristic phases of the intracellular cardiomyocyte potential.^21^ Intracellular measurements were also obtained with branched intracellular nanotube field‐effect transistors (BIT‐FETs), which combine FET detector elements with highly scalable nanotubes extending perpendicularly from the plane of the chip.^22^ BIT‐FET devices have been fabricated with diameters as small as 3 nm and estimated bandwidths up to ≥6 kHz, suggesting minimal invasiveness and the capability to measure rapid neuronal action potentials. An extension of the BIT‐FET is an active silicon nanotube transistor (ANTT) in which the insulated source and drain are defined at the end of the nanotube.^23^ FET‐based approaches are particularly attractive because their nanometer‐scale size allows for higher spatial resolution compared to MEAs. However, a drawback of FETs is that they are less suitable for delivering stimulatory pulses—a prerequisite for many applications—and, in most cases, an open FET used for recording needs to be accompanied by a capacitive‐type stimulation spot.^24^, ^25^, ^26^ In summary, the large variety of different experimental approaches described herein involve complex and nonscalable fabrication processes, require specialized equipment and training, or lack high‐throughput and especially 3D spatiotemporal recording capabilities.

In this study, we exploit stress‐based roll‐up and differential residual stress in nanoscale bilayer constructs^27^, ^28^, ^29^ to create multielectrode shells: self‐folding microgrippers with multiple embedded and individually addressable electrodes. Microscale grippers have previously been fabricated to facilitate encapsulation and optical analysis of single cells.^30^ These grippers have demonstrated biocompatibility for long‐term live cell studies and capability of precise analysis at the single cell level. In contrast to regular 2D substrates, the microgrippers trap cells providing a larger area of firm contact. Consequently, they can function as a shell sensor for cell membrane analyses.^31^ The process of conventional microfabrication and parallel assembly ensures highly parallel and scalable production, which yields a large array of devices that can be operated in a chip‐based format. These cell‐gripping microstructures can be readily patterned with other features such as nanoparticles, 2D layered materials (2DLMs), and adhesive/nonadhesive patches for diverse applications. The designed self‐folding microstructures in this study contain patterned and individually addressable electrodes that are embedded in each of the four arms of the gripper, in order to create a multielectrode shell for live cell stimulation and 3D spatiotemporal recording.

## 2 Results and Discussion

### 2.1 Chip Fabrication

**Figure 1:**
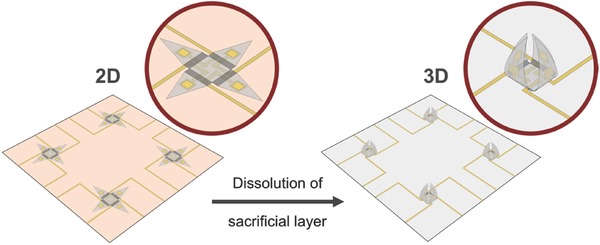
Illustration of the self‐folding 3D multielectrode shell. Self‐folding microgripper structures are patterned on top of a dissolvable sacrificial layer (orange surface). The triangular SiO_2_ panels contain embedded Au electrodes (yellow squares) and interconnects that are connected to outside bond pads, while the residual stress actuated hinges (dark gray rectangles) are composed of a prestressed nanoscale bilayer of SiO/SiO_2_. Upon dissolution of the sacrificial layer, the intrinsic stress is released and the panels with embedded electrodes fold inward.

The concept of the self‐folding, individually addressable multielectrode shell is illustrated in **Figure** **1**. When the shell is in the open state, all electrodes are in a planar configuration. Upon dissolution of a sacrificial layer, the intrinsic differential stress of a SiO/SiO_2_ bilayer is released and the panels with the embedded electrodes self‐actuate and fold toward the middle of the structure. The electrodes in the shell are individually addressable via photolithographically patterned interconnects. The integration of such multielectrode structures with living, electrogenic cells allows for a new approach for cell recording with 3D spatiotemporal control, physically gripping the cells and providing a robust cell‐electrode interface.

We fabricated the devices on a (100) Si wafer coated with 500 nm of thermal chemical vapor deposited (TCVD) SiO_2_. Passivated Au interconnect lines are first patterned using photolithography and subsequently covered with a dissolvable sacrificial layer. Next, a bilayer of SiO/SiO_2_ is e‐beam evaporated on top and a subsequent wet oxide etch defines the general cross‐shape of the multielectrode shell. Passivated Au interconnect lines are extended to each of the four cardinal points of the structure and rigid panels of SiO_2_ are then patterned on top. Finally, the Au interconnects are extended upward again through the panels and the exposed Au surface area serves as the electrode that contacts the cell and transfers any recorded membrane potential back to the bond pads (details of the process flow are in the Supporting Information, together with Figures S1 and S2, Supporting Information).

### 2.2 Shell Folding and Cell Interfacing

**Figure 2:**
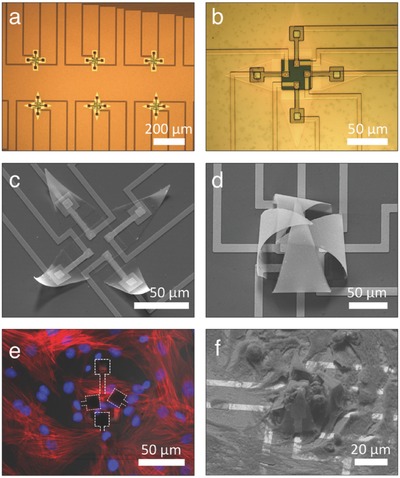
Scanning electron microscopy (SEM) and optical images of an individually addressable, multielectrode self‐folding shell. Optical images showing a) an array and b) a single planar microfabricated precursor with semi‐rigid panels, electrodes, and interconnects. SEM pictures of c) a semi‐closed shell structure, illustrating self‐actuation of the arms and the transition from the open to the closed configuration, and d) a completely closed shell with four individually addressable electrodes. Cardiomyocytes captured within electrode shells imaged using e) confocal fluorescence microscopy showing actin filaments (red) and cell nuclei (blue) with three electrodes wrapping around the cells (dashed white lines), and f) SEM picture showing captured cardiomyocytes after fixation.

Optical images of the individually addressable multielectrode shells are shown in planar and self‐folded form in **Figure** **2**a–d. The fabrication approach leverages planar conventional very‐large‐scale integration (VLSI) approaches which afford significant throughput, fidelity, scalability, and tunability. A typical multielectrode shell chip consisted of multiple shells with various sizes and configurations. In the open configuration, their width varied between 52 and 170 µm panel‐to‐panel, so as to encapsulate few numbers of cells; larger or smaller devices could as well be formed if needed by varying CAD designed optical masks. The shells were designed with square or triangular faces, and the size of the cell contact electrodes ranged from 16 to 144 µm^2^. The majority of shells had electrodes patterned in each of their four cardinal points, but electrodes could alternatively be incorporated in the center at the bottom of the shell, or elsewhere as needed. Additionally, each panel electrode can be individually addressed, as used in our studies, or could be connected to a single bond pad if needed. After the full fabrication flow, the wafer was diced into individual chips that were glued and wire bonded on top of a printed circuit board (PCB). Depending on their thickness, the panels can either fold in a rigid way or display some curvature. For example, by utilizing an ultrathin film bilayer, the stress can also cause each panel to be flexible and curve during folding which accentuates the shell shape. For optimal enclosure of the shell around the cell, and thus optimal cell–electrode contact, we chose to work with such panels that we hypothesize can more readily conform to the cell (Figure 2c,d).

Prior to cell seeding, a 25% copper etchant was added to the cell containment chamber to remove the Cu sacrificial layer. After ≈1 min of etch time, all the Cu is removed except for a tiny sliver underneath each shell panel. This procedure ensures that the cells are minimally exposed to Cu ions, while keeping the panels attached to the substrate. Next, after a thorough rinse step in deionized (DI) water, the chips were sterilized using 70% EtOH, coated with fibronectin and seeded with primary cardiomyocytes. Over time, the cells settle and attach to the substrate while the cell culture medium continues to dissolve the remaining sliver of Cu under the panels. Once the actuation force from the release of the differential stress of the hinges exceeds the adhesive force, the panels are released from the substrate (Movie S1, Supporting Information) and any cell laying on top will be captured by the multielectrode shell (Figure 2e). Based on a time‐lapse experiment in phosphate‐buffered saline (PBS), the pre‐etched panels release after ≈8–10 h.

We validated electrophysiological behavior of the cardiac cell monolayer—and thus also viability—by loading the cells with the Fluo‐4 AM calcium indicator after 3 d in vitro (DIV). Spontaneous and uniform Ca^2+^ transients with corresponding rhythmic contractions could be observed over the entire chip surface (Movie S2, Supporting Information). Simply by changing the focal plane of the microscope, cardiomyocytes contained within the arms of the multielectrode shell could clearly be distinguished from the rest of the monolayer. Contained cells conformed to the shape of the shell while still exhibiting their typical cytosolic Ca^2+^ transients, indicating viable and functional cells with a maintained bioelectrical activity (Movie S3, Supporting Information). Moreover, with each contractile event the electrodes were observed to move in conjunction with the cell, highlighting tight cell–electrode contacts (Movie S4, Supporting Information). Subsequent characterization using scanning electron microscopy (SEM) shows how the structures intimately integrate in the cellular monolayer, with cardiomyocytes growing in between cell‐harboring shells (Figure 2f). Notably, the gripping process does not produce intracellular Ca^2+^ overload or evidence of cell injury.

### 2.3 Interconnect Design and Optimization

**Figure 3:**
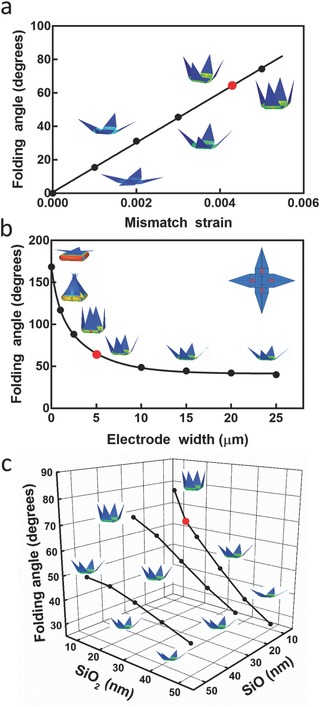
Finite element analysis (FEA) of the folding angle of the multielectrode shell. Graphs showing the variation of the folding angle as a function of a) mismatch strain, b) width of the electrode lines, and c) SiO/SiO_2_ bilayer thickness together with the corresponding shell configuration. The red dots indicate the experimental parameters used for the fabrication: electrode interconnect width 5 µm, SiO/SiO_2_ bilayer thickness 10/15 nm, and strain 0.0043.^30^ More details about modeling, the parameters used, and graphs can be found in the Supporting Information.

Enabling multipoint 3D spatiotemporal electrical sensing, the incorporation of individually addressable embedded electrodes increases the complexity of fabrication and impacts the final folding angles. An optimal device should ensure good contact between the electrodes and the cells, as well as provide enough electrical conductivity and necessary insulation. During design and fabrication, it is important to determine the parameters and materials for optimal function. Finite element analysis (FEA) provides a powerful approach to predict deformations of structures with complex shape. Using FEA, we modeled the effect of key variables, including mismatch strain, interconnect dimensions, and SiO/SiO_2_ bilayer thickness on the folding angle. Details are discussed in the Supporting Information. Overall, the folding angle increases as the mismatch strain increases (**Figure** **3**a); this mismatch strain can be controlled by the thin film deposition conditioning of the SiO/SiO_2_ bilayer. FEA simulations also suggest that the folding angle is sensitive to the interconnect width (Figure 3b). The embedded interconnect at the hinge increases the bending stiffness and thus decreases the folding angle. It is necessary to optimize the dimensions of the interconnects (i.e., width, thickness, and materials) in order to keep a proper folding angle without compromising the conductivity and insulation. Furthermore, film thickness is a tunable parameter during fabrication and has a great influence on the folding angle. We examined how the folding angle changes with different thicknesses of SiO and SiO_2_ in each layer (Figure 3c). In general, larger folding angles are expected as thickness decreases, due to decreasing bending stiffness. Nevertheless, the film needs to be thick enough to maintain the structural integrity and avoid fracture during self‐folding. Together, these analyses provide valuable insights to determine the optimal design and fabrication parameters for panels and interconnects. For the simulations the panels were assumed to be rigid, while the experimentally realized panels in Figure 2c,d are curved, creating the appearance that the folding angle is greater compared to the theoretical value calculated using FEA. However, the experimental fold angle between the substrate and SiO/SiO_2_ bilayer (excluding the panel) is in close agreement with the FEA modeling results.

### 2.4 Extracellular Recordings

**Figure 4:**
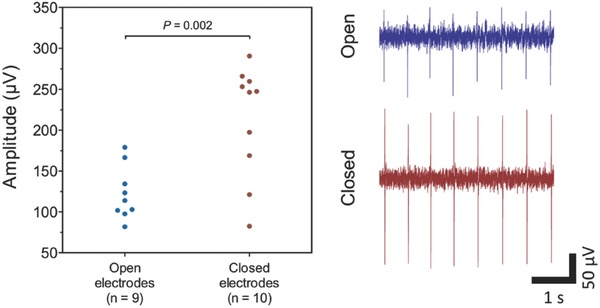
Comparison of signals between closed and open electrodes, all recorded at the same time point from the same chip. Every data point in the plot reflects the averaged peak amplitude of 240 action potentials recorded during a 2 min interval by individual panels. Cardiac action potential amplitudes recorded by closed panels are statistically larger (*p* = 0.002) compared to the open, planar configuration. Example traces are shown on the right.

**Figure 5:**
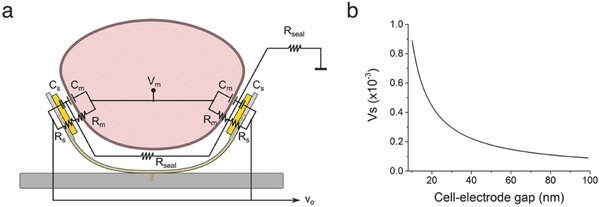
Schematic showing the equivalent circuit during shell recording. a) Illustration of a cell volume inside a deformed multielectrode shell together with the electrical equivalent circuit of the cell–electrode junction. Due to the intrinsic compressive stress of the panels, the electrodes are pushed against the cellular membrane and *R*_seal_ consequently increases. b) Measured output voltage *V*_s_ (here dimensionless) in function of the distance between the cell and electrode, based on the area‐contact model of Joye et al.^32^

Primary cardiac cell signals were recorded after three DIV using a MEA‐headstage with preamplifier. A significant finding was that the signal picked up by electrodes that wrapped around the cell was consistently and approximately twofold higher compared to electrodes that remained in the planar, open configuration (**Figure** **4**). The improved output signal can be attributed to the force exerted by the stressed SiO/SiO_2_ bilayer that pushes the electrodes against the membrane of the captured cell. The gap between cell and electrode will therefore be smaller compared to the conventional situation where the cell is laying on top of the electrode. Hence, the resistance between this gap and the surrounding solution (i.e., the sealing resistance *R*_seal_) is increased, causing less leakage current and consequently a higher measured signal amplitude.^3^ This is schematically represented in **Figure** **5**a, together with the electrical equivalent circuit of the cell–electrode junction in the multielectrode shell. It includes the intracellular membrane potential *V*_M_, the measured signal amplitude or output voltage *V*_o_, the membrane capacitance *C*_M_, the membrane resistance *R*_M_, the electrode capacitance *C*_S_, and the electrode resistance *R*_S_. In order to demonstrate the dependence of the recorded amplitude on the gap between the electrode and cellular membrane, we performed an analytical simulation based on the area‐contact model described by Joye et al. (see the Supporting Information),^32^ which takes into account the spatial distribution of the electrical characteristics in order to describe the electrical properties of the cell–electrode interface at subcellular level. As shown in Figure 5b, the sensed output voltage *V*_s_ approximately doubles when the cell is twice as close to the electrode. Based on our measured signal amplitudes, we therefore assume that the force exerted by the stressed bilayer decreased the cell–electrode distance approximately by half. It would be interesting for future investigation to determine the relationship between the intrinsic stress of the bilayer and measured amplitude, together with other relevant electrogenic cell types such as neurons.

**Figure 6:**
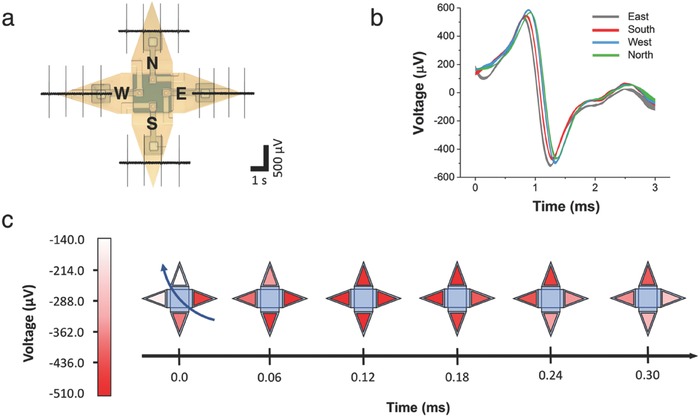
Spatiotemporal live cell recording using individually addressable multielectrode shells. a) Measured extracellular recordings of a single folded shell from each individually addressable electrode enclosing a cardiomyocyte (drawn in the open state to improve comprehension). b) Overlap of averaged action potentials (error bands) recorded by each of the four panels. c) The small delays between the individual traces indicate that the action potential was travelling from southeast to northwest (blue arrow).

The overall design of the individually addressable multielectrode shell allowed simultaneous recordings of every folded electrode located on each of the four cardinal points (**Figure** **6**a). Superpositioning of the extracellular action potentials shows that consistent cell recordings were possible—both in the open and closed shell configuration—and that the multielectrode shell was capable to track action potential propagation in the cells contained within the shell (Figure 6b,c). The measured conduction velocity was 46–50 cm s^−1^; these values match conduction velocities reported for cardiac tissue, and more specifically for ventricular cells.^33^, ^34^, ^35^ These data highlight a significant advantage of an individually addressable multielectrode shell in that it is possible to record 3D spatiotemporal electrical signature from captured cells.

## 3 Conclusion

In summary, a novel and first‐of‐its‐kind multielectrode shell chip has been developed with self‐folding electrodes that bridges the gap from 2D planar recordings to more complex 3D interfacing, allowing parallel readout of all cardinal points of electrogenic cells with higher signal‐to‐noise ratios. The fabrication is done using conventional cleanroom technology allowing cost‐effective, wafer‐level‐based batch processing and future integration with CMOS modules for switching and signal processing. As demonstrated, this device can provide the foundation for the development of new‐generation MEAs where dynamic electrode–cell interfacing and recording substitutes the traditional method using static electrodes. Accordingly, future research effort will have to focus on the next major step of combining these cell‐sized multielectrode shells with microelectromechanical systems (MEMS) in order to have precise control over their position and force, ultimately acting as impactive or even ingressive end effectors. Also the integration of microfluidic layers can be of great interest, which is currently in the scope of our future research and development activities. Finally, by scaling electrode density, highly parallel 3D spatiotemporal electrical maps could be recorded from few or potentially individual cells with micrometer‐scale resolution which would advance our understanding of cellular function, heterogeneity, and response times.

## 4 Experimental Section

*Cell Culture*: Neonatal rat ventricular cardiomyocytes were harvested from 2 d old Wistar rats. Animals were handled in accordance with international (EU Directive 86/609/EEC) and national laws governing the protection of animals used for experimental purposes, minimizing distress during procedures. The use of animals and procedures was approved by the Ethical Committee for Animal Welfare (ECD, Ethische commissie Dierenwelzijn) of KULeuven and Imec. The extracted ventricles were washed in Hank's balanced salt solution (HBSS), followed by overnight incubation at 4 °C in 0.05% trypsin. Next, the tissue was dissociated by adding collagenase for 15 min at 37 °C. Cells were separated through trituration and centrifugation and added to primary cardiomyocyte medium, after which they were pre‐plated to allow for selective attachment of remaining fibroblasts. After counting and a final centrifugation step, a desired concentration of cardiomyocytes was added to cell culture medium and seeded on the substrate.

*Electrophysiological Experiments*: Extracellular action potentials were recorded using a preamplifier with a blanking circuit (MEA1060‐BC‐PA, Multi‐Channel Systems, Reutlingen, Germany) with a gain of 1100, a sampling rate of 50 kHz, and band‐pass filter from 1 Hz to 3 kHz. Each recording session lasted for at least 2 min.

*Fluorescent Imaging and Scanning Electron Microscopy*: Relative fluorescent changes in the intracellular calcium concentration were visualized using the fluorescent marker Fluo‐4 AM (Invitrogen, Belgium), loaded in the myocytes using the cell‐permeant AM ester form. Fluo‐4 was excited at 488 nm and emission signals over 516 nm were collected using an upright Examiner microscope (Carl Zeiss, Belgium). For the actin staining, the cardiomyocytes were fixed in prewarmed 4% paraformaldehyde fixation buffer for 10 min, washed three times with PBS and permeabilized in 0.1% Triton for 5 min. After another PBS wash, an Alexa Fluor 633 Phalloidin solution (Invitrogen, Belgium) was added for 1 h and finally washed one last time in PBS prior to imaging using a Carl Zeiss LSM 780 confocal laser scanning microscope. For SEM imaging of folded shells without cells, the entire Cu sacrificial layer of the sample was etched using APS‐100, subsequently transferred to a 100% ethanol solution and washed at least three times. If cells were cultured on top, samples were first fixed for 10 min using a 4% paraformaldehyde fixation buffer. Next, cells underwent postfixation in a 2% osmium tetroxide (OsO_4_) solution for 2 h, followed by dehydration using a series of increasing concentrations of ethanol (2 × 10 min of 10, 30, 50, 70, and 90%, followed by 3 × 10 min of 100%). Finally, all samples (with and without cells) were dried in a liquid CO_2_ critical point dryer (Automegasamdri‐916B, Tousimis), sputter‐coated with 2 nm of Pt to improve conductance and subsequently imaged using SEM (Nova NanoSEM 200, FEI).

*Statistical Analysis*: Statistical analysis was performed using the GraphPad Prism 5 software. Normality was examined by means of a D'Agostino & Pearson omnibus normality test, and numerical data were compared directly using an unpaired *t*‐test.

## Conflict of Interest

The authors declare no conflict of interest.

## Supporting information

Supplementary

Supplementary

Supplementary

Supplementary

Supplementary
