# Supplementary material for: A Micropatterned Multielectrode Shell for 3D Spatiotemporal Recording from Live Cells
Source: Adv Sci (Weinh). 2018 Jan 4;5(4):1700731. doi: 10.1002/advs.201700731 (PMC5908352; doi:10.1002/advs.201700731)
Supplement: Supplementary file 1 — Supplementary [file ADVS-5-1700731-s002.pdf]

Copyright WILEY-VCH Verlag GmbH & Co. KGaA, 69469 Weinheim, Germany, 2018.

ADVANCED SCIENCE
Open Access

Supporting Information

for *Adv. Sci.,* DOI: 10.1002/advs.201700731

A Micropatterned Multielectrode Shell for 3D Spatiotemporal Recording from Live Cells

Jordi Cools, Qianru Jin, Eugene Yoon, Diego Alba Burbano, Zhenxiang Luo, Dieter Cuypers, Geert Callewaert, Dries Braeken, and David H. Gracias**

Copyright WILEY-VCH Verlag GmbH & Co. KGaA, 69469 Weinheim, Germany, 2017.

Supporting Information

A micropatterned multielectrode shell for 3D spatiotemporal recording from live cells

Jordi Cools, Qianru Jin, Eugene Yoon, Diego Alba Burbano, Zhenxiang Luo, Dieter Cuypers, Geert Callewaert, Dries Braeken, and David H. Gracias**

Note S1. Process flow of the chip fabrication process

The substrate consisted of a (100) bare silicon wafer with 500 nm of thermally grown silicon dioxide (SiO_2) on top. For the electrical wiring that lies underneath each shell, 10 nm of Ti and 40 nm of Au was sputtered and patterned by a standard lift-off process using a combination of lift-off resist LOR 1A and positive photoresist IX845. Each wire (5 µm wide) runs between the central face of each shell and the bond pads. In a second photolithography step, a 100 nm Si_3N_4 layer was deposited to insulate the circuit paths and 50 nm of Cu served as the sacrificial layer. Only the bond pads and central face of the electrode remained exposed. Next, the full wafer was covered with a 10/15 nm SiO/SiO_2 bilayer using e-beam evaporation and the general cross shape was defined by a third photolithography step and subsequent wet etch process in buffered HF. It also defined the vias in the central face that were needed to connect the underlying Au layer to the interconnects in a later stage. After filling the vias with 30 nm of Au, the branching interconnects were patterned to connect these vias to the electrodes on the outer faces of the multielectrode shell. The interconnects consisted of 5 nm Ti and 10 nm of Au, passivated with 15 nm of Si_3N_4. Lastly, after patterning the 200 nm SiO_2 rigid faces, vias were etched and filled with Au to serve as the electrodes that eventually contact the electrogenic cell. After this final process step, the wafer was diced into individual chips, which were glued to a printed circuit board (PCB) by a non-conductive epoxy, cured at 150°C for one hour and subsequently wire bonded. Wires were protected by a layer of biocompatible epoxy (EPO-TEK 353ND-T, Epoxy Technology) and thermally treated at 100°C for several hours. Prior to cell culture experiments, a glass ring lid was glued to the PCB to form the containment volume chamber. Almost the entire Cu sacrificial layer was rapidly etched using a 25% APS-100 copper etchant (Transene, USA), except for a sliver of Cu under the panels to hold them down until the cells were present.

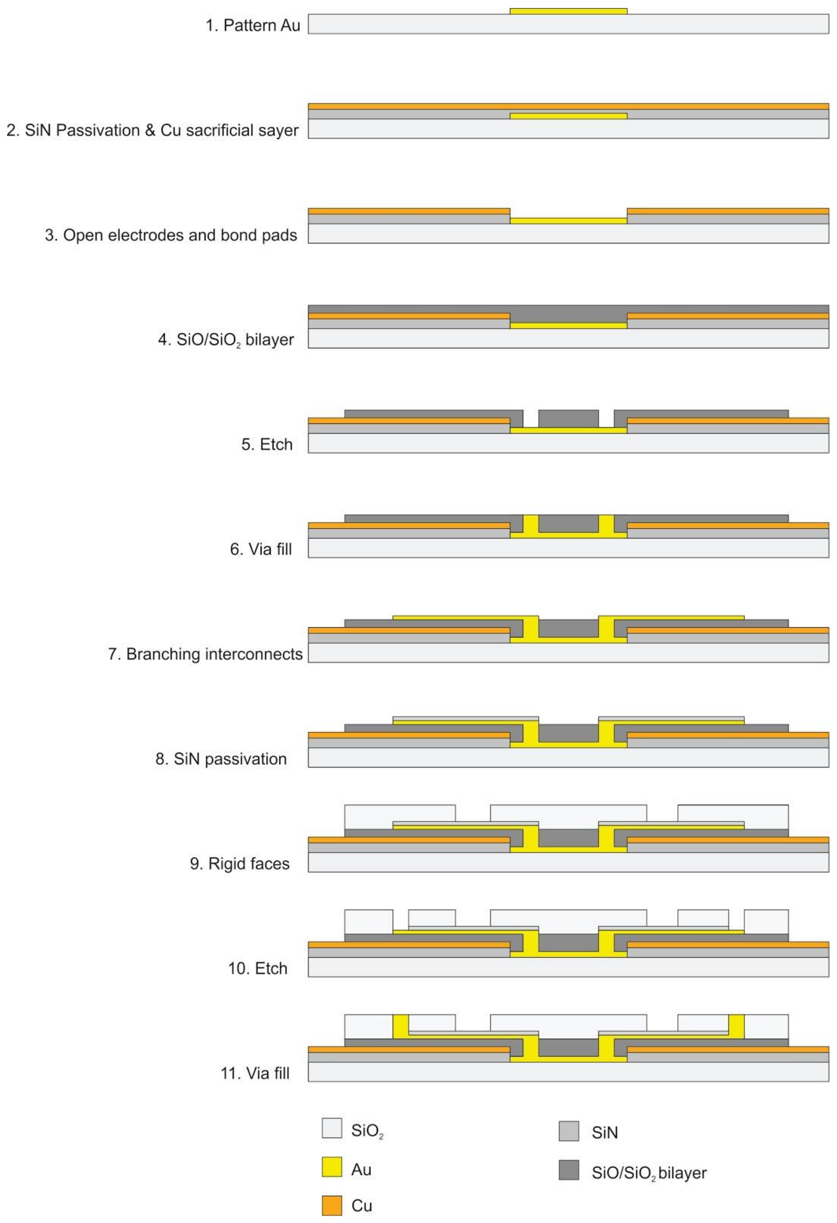

Figure S1. Process flow for the fabrication of the multielectrode shells.

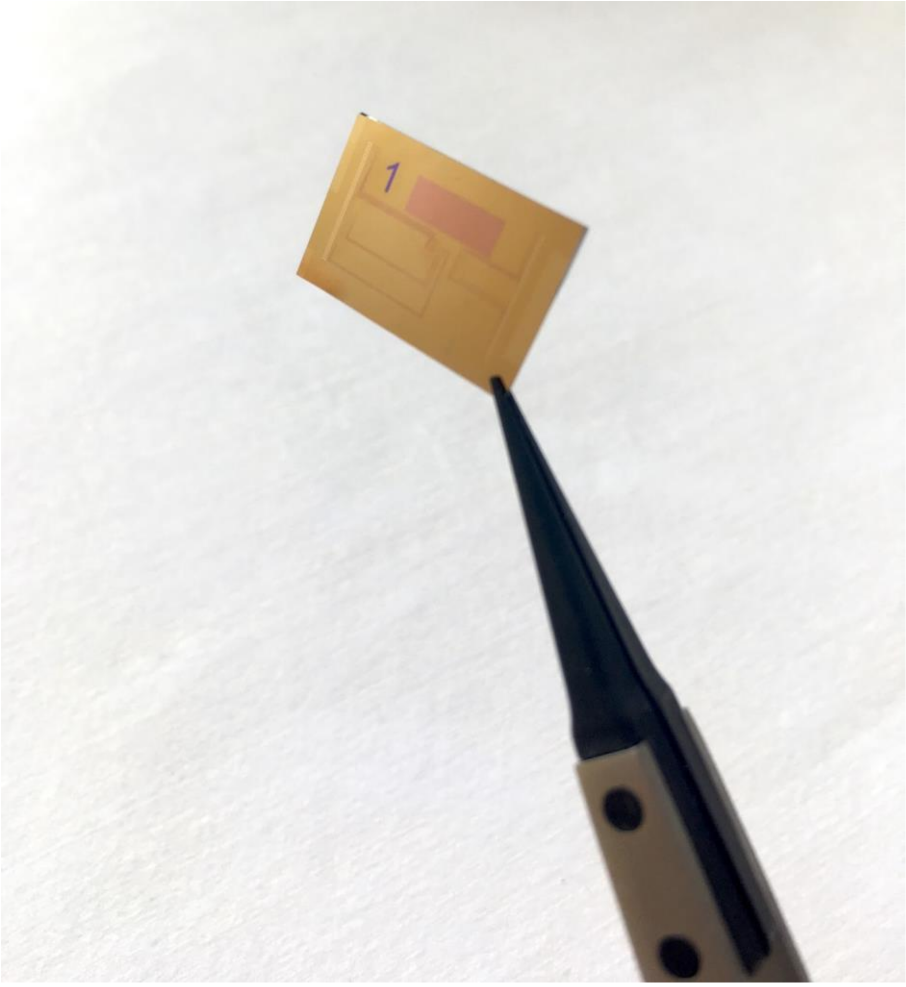

Figure S2. Image of a typical integrated chip after the final fabrication step. The chip dimensions are approximately 2 x 1 cm.

Note S2. Note on yield and fabrication constraints

Using state-of-the-art equipment in imec Belgium's III/V lab cleanroom we were able to fabricate the multielectrode shell platforms with high levels of architectural control. The main difficulties lay in the optimization of every single process step (e.g. resist selection, material film thicknesses, deposition techniques, etch times, etc.). Thus, the fabrication of the platform itself is relatively easy, given the right set of tools and knowledge about general cleanroom work procedures. Consequently, the frequency of folding was relatively high. A reasonable estimate based on our experience with the platform is an average folding ratio of at least 90%.

We attribute any failure to incomplete dissolution of the sacrificial layer at the time of the experiments, and errors in the fabrication process (e.g. lift-off imperfections, defects in the photoresist film due to trapped air bubbles, etc.).

Note S3. Details of the finite element analysis and parameter study

S3.1 Finite Element Analysis

The multielectrode shell folding angle as a function of mismatch strain, interconnect width and bilayer thickness were investigated using a finite element computational model. We used finite element software Abaqus to model the gripper folding process. The inputs include dimensions and estimated stress. The outputs are deformation and stress distribution. The representing multielectrode shell is modeled based on the CAD mask design and fabrication parameters. The device is made of one fixed center, four arms with flexible hinges and rigid segments. The tip-to-tip size of one shell is 120.8 μm. The hinge is 25 μm wide and 9.6 μm long. In the simulation, we assumed that only the hinge is deformable. The deformable hinge was simplified as a composite shell due to the large aspect ratio between lateral dimensions and thickness. Each layer of the composite shell was assigned with corresponding material property and thickness, as listed in Table S1. Simpson integration rules with 61 points was used for each layer.

Table S1. Material properties and dimensions used in the simulation.

Material	Young's modulus (GPa)	Poisson ratio	Thickness at the hinge (nm)
SiO	77	0.2	10
SiO_2	75	0.17	15
Ti	116	0.32	5
Au	79	0.40	10
Si_3N_4	160	0.253	15

Boundary conditions: The center point was set as pinned with no displacement or rotation. The x=0 axis in the center was set to be x-symmetrical so that there is no displacement in x direction and no rotation in y and z direction. The y=0 axis in the center was set to be y-symmetrical so that there is no displacement in y direction and no rotation in x and z direction.

We modeled the mismatch strain by using thermal expansion difference as an equivalent input, since there was no direct available input for mismatch strain from the software. The mismatch strain between SiO and SiO_2 was realized by assigning a different thermal expansion coefficient α to each layer. When applied a temperature field ΔT at the hinge, the hinge undergoes a deformation with different expansions at different layers ($\varepsilon = \Delta T . \Delta\alpha$), which is equivalent to the mismatch strain $\varepsilon = \frac{\sigma_1}{E_1} - \frac{\sigma_2}{E_2}$.

Mesh and step: The deformable hinge was meshed with 38 x 30 structured quadratic elements. The center and the rigid segments were meshed with 40 x 30 structured quadratic elements. Increasing the mesh size further did not change the results. The entire process was simulated by a static model with step length from 10^{-15} to 10^{-2}. Nonlinear geometry deformation is also considered.

S3.2 Effect of interconnects design and optimization of fold angles

Previously, we have shown that SiO/SiO_2 bilayers can be used to create self-folding devices due to release of residual stress. However, significant optimization was required to create electrodes on the device. As discussed in the fabrication process, the interconnects at the hinge consist of a conductive layer for electrical connection and an insulation layer to prevent short-circuit with surrounding environment. The incorporation of electrodes increases the bending stiffness, i.e. decreases the folding angle. It is necessary to optimize the design of

interconnects (i.e. width, thickness, materials) in order to keep a proper folding angle without compromising the performance.

The interconnects consist of 5 nm Ti for adhesion, 10 nm Au for conductivity, and 15 nm Si_3N_4 for insulation. All the thicknesses are at the lowest possible values to yield a good folding angle while still ensure their functions. Keeping the thicknesses constant, we have performed FEA to study how different numbers and widths of the interconnects affect the final folding angles. Since the width of the entire hinge is 25 μm, we varied the width of the interconnect from 2.5 μm to 5 μm. We have also varied the number of interconnect from 0 to 2. **Table S2** shows the illustration of interconnects before folding and FEA simulation results after folding. We found that increasing width and numbers of electrodes can greatly reduce the folding angle as expected, due to the increased bending rigidity. The folding angle as a function of interconnect width are plotted in Figure 3C. From these analysis we concluded that adding the interconnects and electrodes would decrease the folding angles, and one need to rationalize the design to ensure the best electrical performance while maintain an appropriate folding angle.

**Interconnect design**	1x<br>2.5 μm wide	1x<br>5 μm wide	2x<br>each 2.5 μm wide	2x<br>each 5 μm wide
**FEA design before folding**	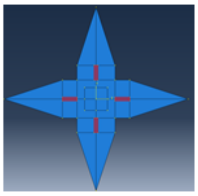	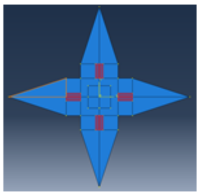	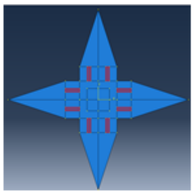	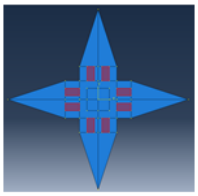
**FEA Results after folding**	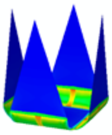	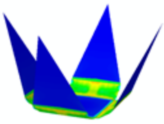	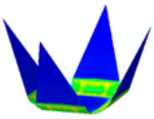	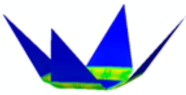

Table S2: Illustration of interconnects before folding and FEA results after folding.

Note S4. Details of the analytical simulation of the cell-electrode interface

Our analytical simulation is based on the area-contact model of Joye *et al.*,[1] where the potential which is sensed by the amplification stage (V_s) is expressed as:

$$V_s(s) = \frac{\pi(1 + r_{ct}(sc_{dl})^n)}{sC_{load}r_{ct} + R_{el}^2\pi(1 + r_{ct}(sc_{dl})^n)}\left[R_{el}^2 + \frac{e^{a(R_{ce}^2 - R_{el}^2)} - e^{aR_{ce}^2}}{a}\right]V_M(s)$$

where r_{ct} is the charge transfer resistance (30 Ωcm^{-2}), c_{dl} the double layer capacitance (432 μF cm^{-2}), C_{load} the load capacitance (10 pF), R_{el} the electrode radius and *n* an empirical factor between 0 and 1 representing surface irregularities. V_s is dimensionless.

Note S5. Additional notes on cell recording experiments

S5.1 Note on biocompatibility and number of cell experiments

In this study the Cu sacrificial layer was removed by using the Cu etchant APS-100. The chip was then thoroughly rinsed to remove any excess etchant, followed by sterilization, and coating with fibronectin for cell adhesion. The entire process ensures no etchant is in contact with cells and cells are in a favorable environment. It should be noted that while e.g. Cu minerals and metallic Cu are largely insoluble at physiological pH, these compounds may behave quite differently in physiological fluids. The cell medium contains a variety of additives including inorganic salts, amino acids, peptides, proteins, carbohydrates. Moreover, the temperature of the cells is always kept at 37°C in an incubator. All these factors greatly enhance the solubility of Cu.[2–5] Alternative sacrificial layers such as germanium, which can be released by water or PBS, have been demonstrated in previous research, and can be adopted if needed. The green fluorescence changes reflect spontaneous cytosolic Ca^{2+} transients and indicate viable cells with a maintained bioelectrical activity. These findings strongly suggest the presence of functional gap junctions and that the impact of the platform on cell viability and functionality can be considered minimal. Cardiomyocytes were shown to remain viable for at least ten days in culture and replacement of the cell medium had no detectable effects on entrapped and non-entrapped cells.

For all cell experiments presented in the paper (excluding the number of chips used for optimization of (1) the fabrication process flow, (2) sacrificial layer removal try-outs, and (3) initial biocompatibility experiments) we investigated approximately 10 multielectrode shell platforms which gave adequate results for the experiments intended.

S5.2 Note on the duration of cell recording using the platform

We note that all recordings in the manuscript were performed at 3 DIV when the remaining sliver of sacrificial layer is completely dissolved and cardiomyocytes form a confluent

monolayer that exhibits synchronized contraction. Compared to 5 or 7 DIV, the quality of the recorded signals was far superior at 3 DIV, suggesting that over time there is some degradation of the multielectrode shells in the culture medium. This could be due to the slow dissolution of the SiO/SiO_2 bilayer, as investigated in Malachowski *et al.*, thereby reducing the force exerted by the bilayer on the cell.[6]

In theory, alternate combinations of bilayer and sacrificial materials which are less prone to degradation could be used. We opted for SiO/SiO_2 bilayers because strict material constraints are imposed at imec's cleanroom due to sharing of instrumentation with a CMOS line, and because the combination of SiO/SiO_2 was previously already successfully implemented.

S5.3 Note on cell dimensions and number of cells that can be captured in one device

We note that our multielectrode shell chip consisted of multiple shells with various sizes and configurations. In the open configuration, their width varied between 52-170 μm panel-to-panel, so as to encapsulate few numbers of cells. Given cell dimensions of cultured neonatal rat myocytes of approximately 70 μm by 70 μm, some shells should be able to capture one single cell, thus allowing single-cell recording and analysis. However, we deliberately avoided making the statement that this is a single cell recording device as we could never be entirely sure about single cell encapsulation. For example, cardiomyocytes can be stained using calcein AM or Fluo-4 AM and subsequently investigated under the microscope, but it is difficult to discriminate one cell from another (especially because cardiomyocytes in culture may also form multilayers) and additionally primary rat cardiomyocytes are known to be multinucleated. For rodents, the conversion of cardiomyocytes from a mononucleate to binucleate phenotype happens in the early postnatal period,[7] i.e. the time when cells were isolated. Staining the cell nuclei with DAPI or Hoechst dyes is therefore not an option to assess the exact number of cells captured by the shell under investigation.

S5.4 Note on measured signal amplitudes between open and closed electrode configurations

It should be noted that the individually displayed action potential values in Fig. 4 were obtained at the same time point from the very same chip. A comparison of action potentials before and after cell gripping from the same shell proved to be impractical: As discussed previously, prior to cell seeding, the sacrificial layer was removed except for a tiny sliver underneath each shell panel to keep them in place. Over time, this remaining sliver of copper dissolves in the cell medium. Cultured cardiomyocytes, however, need at least three days *in vitro* to form a confluent monolayer that exhibits synchronized contraction, and by this time the tiny sliver of sacrificial layer will be long dissolved in the warm cell medium. Hence, it was not possible to record from a confluent cell layer on open and subsequently closing electrodes. We also deliberately avoided to make the comparison with electrodes patterned at the planar bottom of the shell structure, as it is important that control electrodes underwent the same fabrication process steps as the self-folding ones, which is not the case in this study. While electrodes patterned at the planar bottom of the shell structure require only a few basic process steps, the self-folding electrodes require many more steps will consequently have an effect on the quality of the signal, recorded noise levels, and stimulation efficiency.

For this reason, we simultaneously measured the action potentials of multielectrode shell structures in the planar vs. the folded configuration, where all electrodes will have similar response characteristics, and subsequently checked using a bright field microscope and Fluo-4 AM fluorescent imaging which panels unmistakably (1) closed, (2) remained open, and (3) had cells inside or on top, respectively. Such identification can be done relatively easy, as shown in the Supplementary Movie S2, S3 and S4.

Note S6. Snapshots and brief description of supplementary movies

S6.1 Movie S1

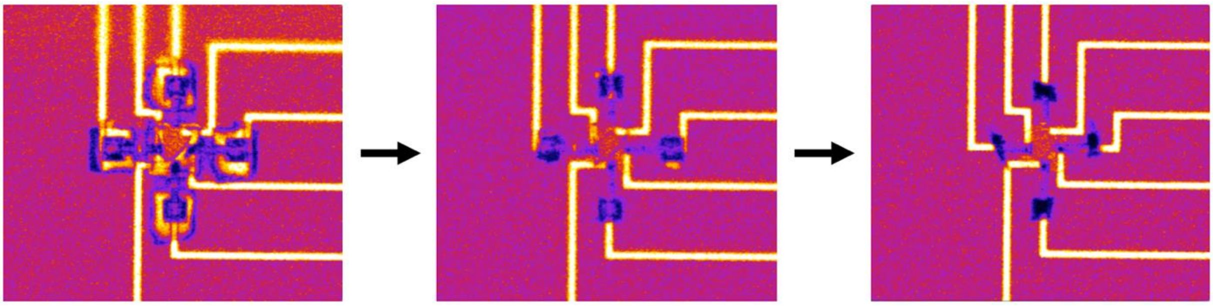

Description: Self-folding of a microgripper structure with electrodes embedded within each of the four panels. Upon dissolution of the sacrificial layer, the intrinsic differential stress of the SiO/SiO_2 bilayer is released and the panels fold towards the middle of the structure.

S6.2 Movie S2

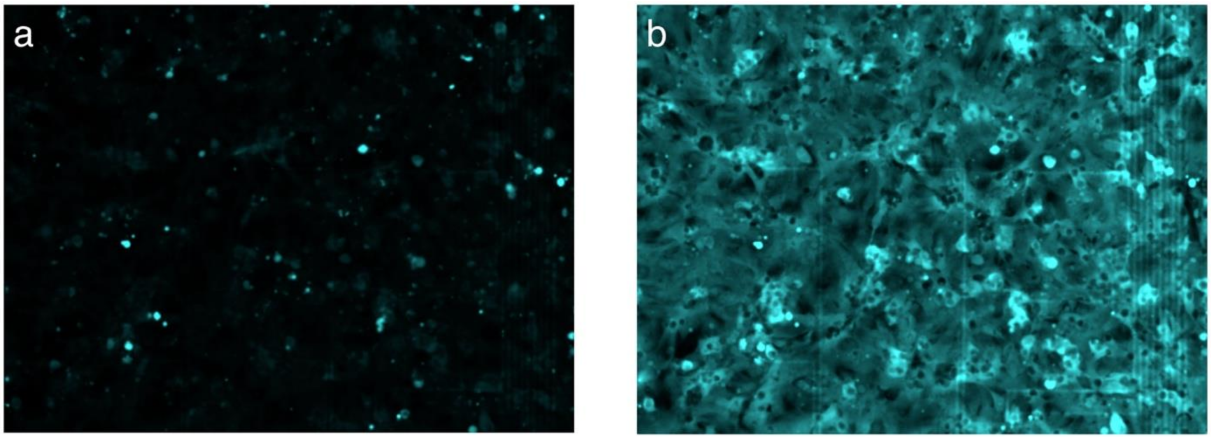

Description: Video showing primary cardiomyocytes cultured on top of an array of multielectrode shells, loaded with the fluorescent marker Fluo-4 AM, a calcium indicator that exhibits (b) an increase in fluorescence upon binding intracellular Ca^{2+} (i.e. with every contraction of the cellular monolayer).

S6.3 Movie S3

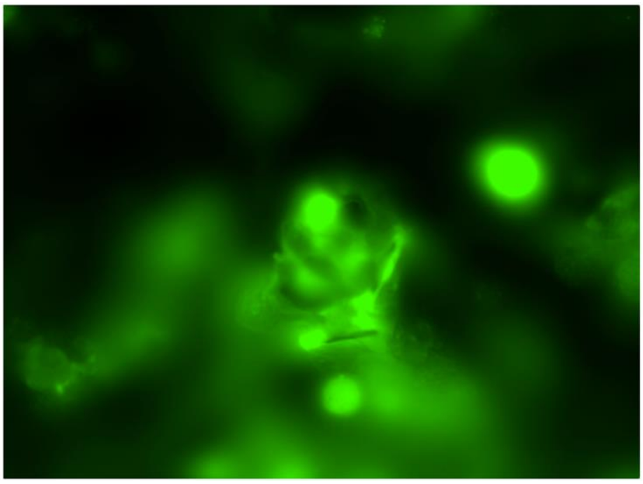

Description: Primary cardiomyocytes loaded with the Fluo-4 AM calcium indicator encapsulated within the multielectrode shell. Contained cells clearly conform to the shape of the shell while still exhibiting their typical cytosolic Ca^{2+} transients, indicating viable and functional cells with a maintained bioelectrical activity.

S6.4 Movie S4

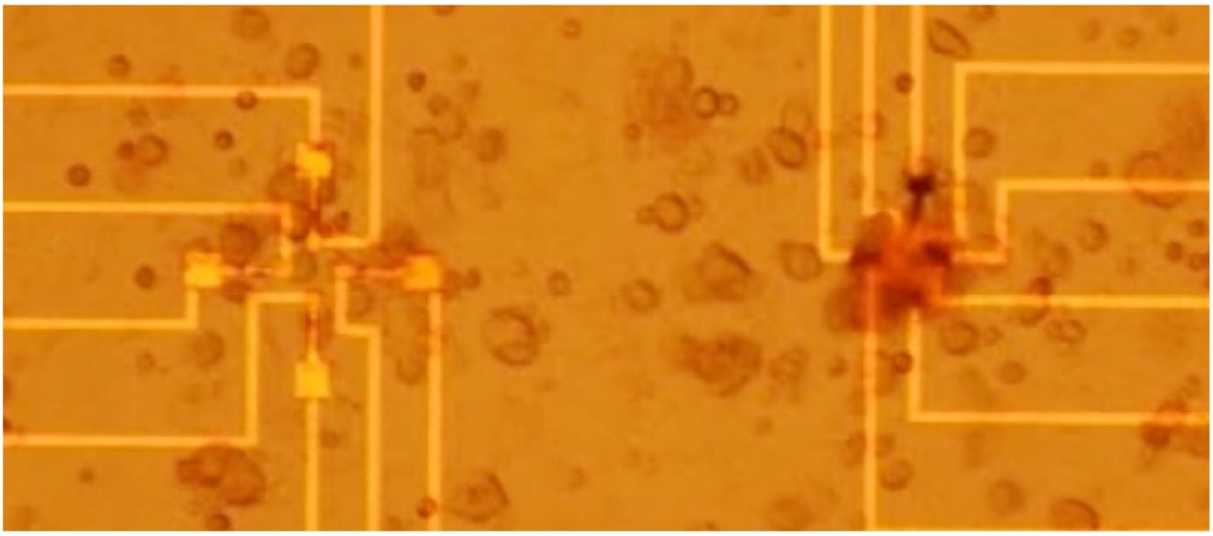

Description: Open (left) and closed (right) multielectrode shell configuration with cultured primary cardiomyocytes. In the closed configuration the arms of the gripper electrodes moved in conjunction with the cell contraction, highlighting tight cell-electrode contacts.

Supplementary References

[1] N. Joye, A. Schmid, Y. Leblebici, *Neurocomputing* **2009**, *73*, 250.

[2] D. P. Graddon, L. Munday, *J. Inorg. Nucl. Chem.* **1961**, *23*, 231.

[3] D. C. Bassett, I. Madzovska, K. S. Beckwith, T. B. Melo, B. Obradovic, P. Sikorski, *Biomed. Mater*. **2015**, *10*, 15006.

[4] L. Song, M. Connolly, M. L. Fernández-Cruz, M. G. Vijver, M. Fernández, E. Conde, G. R. de Snoo, W. J. G. M. Peijnenburg, J. M. Navas, *Nanotoxicology* **2014**, *8*, 383.

[5] L. Li, M. L. Fernández-Cruz, M. Connolly, M. Schuster, J. M. Navas, *J. Nanoparticle Res.* **2015**, *17*.

[6] K. Malachowski, M. Jamal, Q. Jin, B. Polat, C. J. Morris, D. H. Gracias, *Nano Lett.* **2014**, *14*, 4164.

[7] F. Li, *J. Mol. Cell. Cardiol.* **1996**, *28*, 1737.
